# A Conformer-Based Time–Frequency Decoupling Network for Pig Vocalization Behavior Classification

**DOI:** 10.3390/ani16091337

**Published:** 2026-04-27

**Authors:** Jianping Wang, Yuqing Liu, Siao Geng, Feng Wei, Haoyu Wu, Yuzhen Song, Yingying Lv, Shugang Li, Qian Li

**Affiliations:** 1 School of Computer Science and Technology, Henan Institute of Science and Technology, Xinxiang 453003, China; liuyuqing@stu.hist.edu.cn (Y.L.); wuhaoyu@stu.hist.edu.cn (H.W.); reachern@hist.edu.cn (Y.S.); lvyingying@hist.edu.cn (Y.L.); lishugang@hist.edu.cn (S.L.); liqian@hist.edu.cn (Q.L.); 2 School of Foreign Studies, Henan Agricultural University, Zhengzhou 450002, China; chester@stu.henau.edu.cn; 3 School of Computing, Xinxiang Vocational and Technical College, Xinxiang 453006, China; weifeng@xxvtc.edu.cn

**Keywords:** pig behavior, behavioral vocalizations, acoustic monitoring, vocalization classification, precision livestock farming, ATF-Conformer

## Abstract

Continuous monitoring of pig behavior is essential for timely health management and welfare assessment in commercial production systems. Although vision-based methods have been widely studied, their practical application in commercial barns is often limited by variable lighting, frequent occlusion, and high stocking density. Acoustic sensing offers a non-contact alternative that is independent of lighting conditions; however, reliable behavior classification from pig vocalizations remains challenging in commercial environments because of background noise and temporal variability in sound patterns. In this study, an attention-guided acoustic framework, termed ATF-Conformer, was developed for pig vocalization classification under farm conditions. A five-class vocalization dataset was collected from finishing Landrace pigs and multiparous sows on a commercial farm, including cough, scream, estrus, feeding, and normal behavior sounds. The proposed framework combined spectrogram denoising with interactive attention to enhance behavior-related acoustic information, while a time-frequency-decoupled Conformer encoder was introduced to improve feature representation under noisy conditions. Final classification was performed using mask-based temporal pooling with an additive angular margin Softmax objective. In five-fold grouped cross-validation, ATF-Conformer achieved an accuracy of 97.34% ± 0.42 and outperformed several existing acoustic models across multiple evaluation metrics. A similar accuracy of 97.38% was obtained on an independent test set, indicating stable performance across datasets. These results suggest that the proposed method can support continuous, non-invasive pig vocalization-based behavior monitoring and may assist farm owners or workers in pen-level screening of frequent cough or abnormal vocal events, thereby supporting targeted on-site inspection in precision livestock farming.

## Simple Summary

Keeping track of pigs on commercial farms is important for finding health and welfare problems as early as possible. However, watching pigs with cameras is not always reliable because animals may be blocked from view, barns can be crowded, and lighting conditions often change. Pig sounds provide another way to understand what is happening in the pen and can be collected both day and night. In this study, we used sound recordings from a commercial farm to identify five common types of pig sounds: cough, scream, estrus, feeding, and normal behavior sounds. The results showed that these sounds could still be distinguished accurately in a noisy barn environment. This suggests that sound-based monitoring could be a useful tool to help farmers detect abnormal conditions earlier and support daily pig management.

## 1. Introduction

In modern agricultural systems, pork is one of the major sources of global dietary protein, making the stability of pork supply a crucial component of food security and agricultural economies [1]. The rapid expansion of intensive and large-scale pig production has significantly increased stocking density, which, while improving production efficiency, has also led to higher risks of respiratory disease transmission [2] and a greater prevalence of stress-related behavioral disorders [3]. These health and welfare challenges not only affect growth performance and reproductive efficiency but also lead to significant economic losses and increased production risks [4]. As a result, there is a growing demand for intelligent monitoring systems that enable early detection of abnormal behaviors through continuous sensing at the individual level and informed management at the group level, thus supporting data-driven precision livestock farming and smart husbandry practices [5]. In line with this, precision livestock farming is gaining attention for its potential to improve swine welfare [6]. Smart farming and artificial intelligence (AI) play a crucial role in ensuring that animal welfare is prioritized in modern agricultural practices [7]. Additionally, the use of pig sound analysis has emerged as an important tool for measuring welfare in smart agricultural systems [8].

Conventional pig monitoring still relies largely on routine inspections and visual assessment by farm personnel, which can be subjective and are sensitive to lighting conditions. Recently, computer vision has been applied to pig identification and behavior management, including multi-scenario behavior recognition with YOLO-based models [9], dynamic counting and tracking [10], long-term video tracking methods for group-housed pigs [11], face recognition using lightweight networks [12], and small-object detection for vulvar region identification during estrus [13]. However, vision-based systems typically require stable lighting and unobstructed views, and their performance often degrades under commercial conditions such as low-light periods, high stocking density, and severe occlusion, limiting their suitability for continuous and reliable monitoring.

Pig vocalizations provide a non-invasive and lighting-independent source of behavioral and physiological information and have therefore attracted increasing attention for on-farm monitoring [14]. Specific vocalization types are linked to distinct welfare and health risks. Estrus-related sounds are associated with reproductive management and are often investigated using vision-based or multimodal approaches [15]. Coughing is a key marker of respiratory disease and has mainly been studied as a single-symptom detection task [16]. Screams are commonly related to pain, acute stress, or aggressive interactions and are therefore relevant for welfare assessment [17]. Nevertheless, most existing studies still focus on specific vocalization types or single-task scenarios, whereas unified multi-class recognition within a single acoustic framework remains insufficiently explored [18].

In field situations, a fusion-based feature representation based on convolutional neural networks has been developed for pig cough recognition [19]. Additionally, heterogeneous fusion of biometric and deep physiological features has been shown to improve the accuracy of porcine cough recognition [20]. Vocal-type classification has been proposed as a tool to identify stress in piglets under on-farm conditions [21]. These studies indicate that existing pig sound recognition systems still mainly focus on task-specific problems such as cough detection, stress-related vocal-type classification, or estrus-associated monitoring, while the number of target classes is usually limited and the recording conditions are often relatively homogeneous [22]. As a result, their applicability to unified multi-class recognition under realistic commercial-farm acoustics remains limited, particularly when multiple behavior-related sound classes must be recognized under heterogeneous barn conditions.

Among the behavior-related acoustic events encountered in commercial barns, feeding sounds are particularly challenging to extract and classify. In practical pig-house environments, non-stationary noise from mechanical equipment, feeding systems, and human activity can obscure discriminative acoustic cues [22]. Unlike coughs or screams, feeding-related sounds are typically dominated by low-frequency, continuous chewing and friction components and have less distinct temporal boundaries, making them easily confounded with ventilation noise, feeder contact, pen friction, and other background activities [23]. Feature-fusion strategies have been explored to improve acoustic robustness [24]. Continuous acoustic event modeling has also been investigated in complex piggery environments [25]. In addition, joint enhancement of spectrogram and speech-related representations has been used to improve pig-sound recognition [26]. However, feeding-related acoustic patterns have received relatively limited attention as an explicit recognition target in pig sound analysis [18]. This makes feeding sounds an important but underexplored category in unified pig vocalization classification under practical farm conditions. This further underscores the lack of a unified and noise-robust acoustic framework for practical multi-class pig sound recognition in commercial barns.

Although deep learning models show promise in controlled or semi-controlled settings, performance often deteriorates in real barns due to noise, reverberation, overlapping events, and strong background similarity between behavior categories. Therefore, a unified and noise-robust acoustic framework is still needed to reliably recognize multiple behavior-related vocalizations under realistic commercial-farm conditions.

To address this gap, we propose ATF-Conformer as a unified acoustic framework for five-class pig vocalization recognition under realistic commercial-farm conditions. Unlike previous systems that mainly target isolated sound categories, the proposed method jointly models cough, scream, estrus, feeding, and normal behavior sounds within a single framework. In addition, front-end spectral gating and multidimensional interactive attention are introduced to suppress noise-dominated components and emphasize behavior-relevant acoustic regions, while the time–frequency decoupled Conformer encoder is designed to reduce spurious temporal–spectral correlations induced by non-stationary barn noise. In this way, the proposed framework is intended to improve robustness under practical farm acoustics and to better address underexplored categories such as feeding-related sounds in unified multi-class vocalization recognition.

The primary objective of this study was to develop and evaluate a robust acoustic framework for multi-class pig vocalization recognition under commercial farming conditions. A real-farm vocalization dataset comprising five sound classes (*n* = 5268) was constructed, and a strict group-wise evaluation protocol was applied to ensure realistic generalization assessment. This design was intended to reduce overoptimistic estimation caused by source-related overlap and to better reflect practical barn acoustic conditions. Our results showed that, under this group-wise evaluation setting, a time–frequency decoupled Conformer encoder with asymmetric inductive bias improved robustness to noise-induced time–frequency entanglement in barn soundscapes. Moreover, integrating spectrogram gating, attention mechanisms, and mask-based temporal pooling enabled reliable classification across multiple evaluation scenarios. This study provides a methodological basis for practical acoustic-based pig behavior monitoring and supports the application of sound analysis in precision livestock farming.

## 2. Materials and Methods

### 2.1. Dataset

#### 2.1.1. Data Collection

All audio recordings in this study were conducted in accordance with the guidelines for the ethical review of laboratory animal welfare and were approved by the Animal Ethics Committee of Henan Institute of Science and Technology (Approval No. LLSC2025107). Data were collected from 10 March 2025 to 9 June 2025 at Yuanyang Mufengyuan Ecological Family Farm in Henan, China, over a total of 92 days. The farm housed approximately 380 pigs in total. Routine recordings in this study primarily covered five independent finishing pens and involved 50 healthy group-housed Landrace finishing pigs aged 4–6 months and weighing approximately 80–110 kg. Each pen measured 15.2 m^2^ (4.0 m × 3.8 m) and housed 10–12 pigs, corresponding to an average floor space of approximately 1.3–1.5 m^2^ per pig. During recording sessions, ambient background noise levels were approximately 60–70 dB(A) in the recording areas, estimated from spot measurements near the pens, and indoor temperatures were maintained between 18 and 24 °C. Detailed environmental conditions are provided in the Supplementary Materials (Table S1).

**Figure 1:**
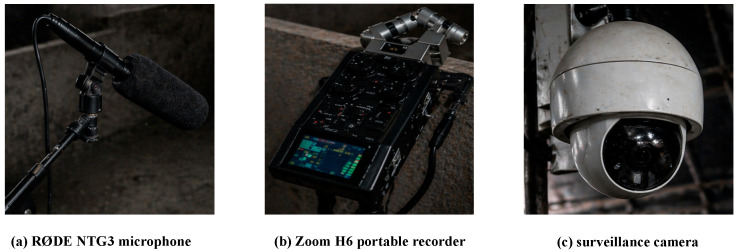
Recording and monitoring devices used during data collection in the commercial pig barn.

Field acquisition was performed using three auxiliary recording units, each consisting of one directional microphone (RØDE NTG3, RØDE Microphones, Sydney, Australia), one portable recorder (Zoom H6, Zoom Corporation, Tokyo, Japan), and one monitoring device. Figure 1 shows representative examples of the devices used during data collection, namely the RØDE NTG3 shotgun microphone, the Zoom H6 portable recorder, and the surveillance camera. The monitoring devices were used only to assist on-site observation and annotation and were not used as model inputs.

Audio signals were captured at a sampling rate of 16 kHz with 16-bit quantization. Automatic gain control was disabled to preserve the integrity of acoustic features, and all signals were stored as monaural, uncompressed WAV files. These recording units were sequentially rotated across the five finishing pens. During each recording session, the microphone was mounted on a fixed bracket approximately 1.2 m above the floor and oriented toward the center of the target pen to improve the stability of collective vocalization capture. Each pen was recorded for 4 h per session, and recordings were conducted during morning, noon, and evening periods to maximize coverage of naturally occurring vocal events during behaviorally active time windows.

**Figure 2:**
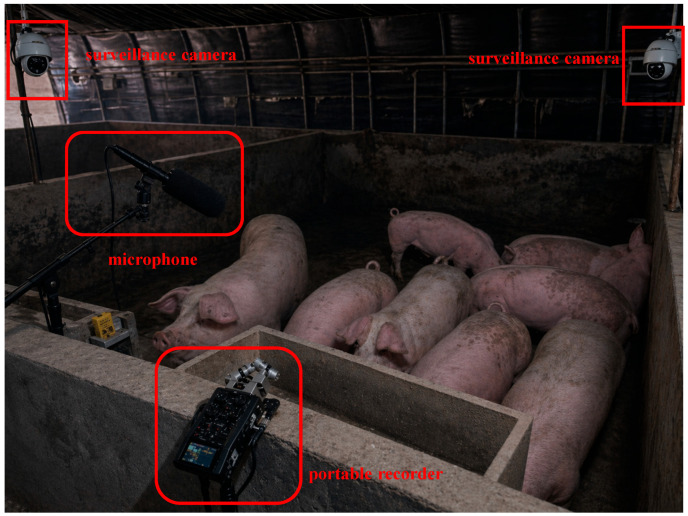
Representative view of the real-farm recording environment in the commercial pig barn.

To capture diverse behavioral scenarios under realistic barn soundscapes, recordings were conducted in two functional areas of the farm. Routine recordings from the finishing area were used to collect scream, feeding, and normal vocalizations under natural barn conditions. Figure 2 presents a representative view of the real-farm recording environment. During data collection, the portable recording setup was temporarily mounted on a fixed bracket and sequentially redeployed across pens, thereby introducing natural variability in behavioral patterns and background noise across recording sessions.

Estrus-related vocalizations were recorded separately in the breeding management area. Multiparous sows were continuously monitored by trained personnel, and once estrus was confirmed, targeted recordings were immediately initiated under standard breeding-stall conditions. Although estrus samples were obtained from breeding stalls rather than finishing pens, all recordings were conducted within the same farm facility and under comparable housing conditions, including similar ventilation and feeding systems. Nevertheless, this strategy may introduce potential recording-area confounds, whereby a model could partially exploit location-specific background cues. To assess this risk, a background separability analysis was performed using non-event segments extracted from long-duration recordings after excluding all labeled vocalization events. Using the same 80-dimensional log-Mel spectrogram features as the main pipeline and grouped cross-validation to prevent session-level leakage, a linear classifier achieved a balanced accuracy of 54.2% (AUROC = 0.56) when discriminating between finishing-pen and breeding-stall background segments, indicating only marginal separability between recording environments.

In addition, all targeted close-range cough samples were constrained by pig-wise mutual exclusion during data partitioning, ensuring that cough instances from the same individual never appeared in both training and evaluation sets.

Because cough events are relatively rare in routine barn recordings, we adopted a dual-scenario acquisition strategy to improve sample coverage while preserving ecological validity. In the final dataset, 26% of cough samples were obtained from long-duration group-housing recordings, whereas the remainder were collected through supplementary close-range recordings of a small number of symptomatic pigs.

Raw audio recordings were processed using a two-stage segmentation pipeline. Continuous recordings were first segmented into 5–10 s clips for event localization and preliminary labeling, followed by trimming into 1–2 s event-centered segments used as final samples. Initial annotations were performed by six experienced farm staff members based on husbandry records, on-site observations, and monitoring information, and were subsequently reviewed by three licensed veterinarians to resolve ambiguous cases and improve labeling accuracy. Disputed samples were discussed until consensus was reached; clips that could not be confidently assigned were excluded from the final dataset. Final annotations with precise timestamps were exported in TextGrid format to support reproducible model training and evaluation.

In this study, a final acoustic dataset was constructed to represent five key pig behavioral categories: cough, scream, estrus, feeding, and normal behavior sounds. The five categories were selected because they cover behavior- or management-related acoustic events of practical relevance to pig health, welfare, reproduction, feeding activity, and routine barn-state monitoring under commercial-farm conditions. Table 1 summarizes the dataset composition, including the number of samples per category, the average duration of input segments, and the corresponding dominant energy frequency bands.

To improve the readability of the dataset description, a concise summary of recording region, class source, close-range acquisition, grouping strategy, and pig-wise exclusion is provided in Table 2.

As summarized in Table 2, the five behavioral vocalization categories were collected from different recording regions and acquisition scenarios, and their partitioning strategies were designed to reduce potential information leakage arising from shared recording conditions or individual-specific effects.

#### 2.1.2. Data Characterization and Feature Analysis

To quantify acoustic differences among behavioral categories and assess their separability in the time–frequency domain, log-Mel spectrograms were extracted from audio samples representing the five target behaviors. This representation applies a nonlinear frequency scaling based on perceptual auditory principles, allowing effective characterization of broadband energy variations in transient events as well as the spectral structure of sustained vocalizations. As a result, log-Mel spectrograms are widely adopted in animal vocalization modeling and classification. An example is shown in Supplementary Figure S1.

**Figure 3:**
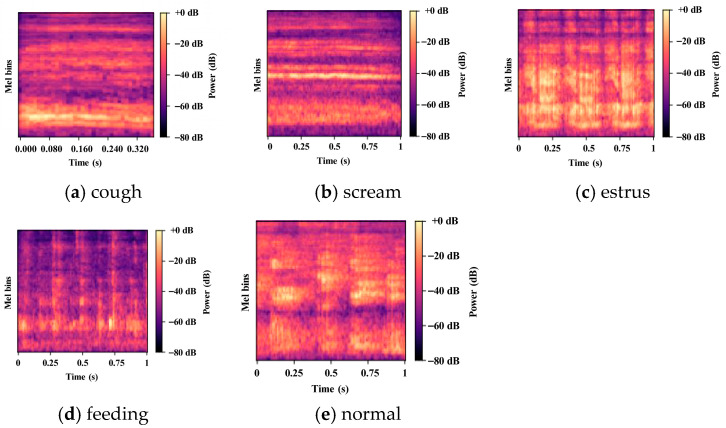
Representative log-Mel spectrograms of five pig behavioral sound categories: (**a**) cough, (**b**) scream, (**c**) estrus, (**d**) feeding, and (**e**) normal behavior sounds.

As illustrated in Figure 3, the five behavioral categories exhibit relatively stable and class-specific patterns in terms of energy distribution, temporal extent, and spectral texture in the Mel spectrogram domain. In this study, sound categories were defined based on a combination of on-site behavioral observations, farm management records, and characteristic acoustic signatures.

Cough sounds typically manifest as short-duration broadband transients with energy concentrated in the mid-to-high frequency range, appearing as spike-like structures in the spectrogram. Screams are characterized by longer durations and higher energy levels, with pronounced high-frequency components forming dense and continuous spectral patterns. Estrus vocalizations often display rhythmically repetitive temporal structures, with energy primarily distributed in the mid-frequency range and exhibiting periodic enhancement along the time axis. Feeding sounds were extracted during active feeding periods and are dominated by low-frequency, continuous chewing or friction components, resulting in rougher spectrogram textures with persistent but non-stationary energy bands. Normal behavior sounds were defined as routine barn acoustic segments recorded from the finishing area outside active feeding periods and in the absence of clearly identifiable cough, scream, or estrus vocalizations. This category was intended to represent typical daily background soundscapes under regular husbandry activities, including low-intensity movement, pen contact, and ambient barn noise, but excluding segments dominated by sustained feeding-related chewing/friction sounds or any salient abnormal vocal event. In this way, the normal class was treated as a controlled reference category for ordinary barn acoustics rather than as a residual class containing all non-target sounds.

These observations suggest that different pig behaviors exhibit distinct and class-specific structures in the time–frequency domain that are amenable to learning. To reduce the influence of farm-related noise and recording-condition variability on acoustic feature extraction, a unified input construction pipeline was employed, including signal normalization and silence trimming. In addition, spectrogram gating was incorporated at the front-end of the ATF-Conformer framework to attenuate noise-dominated components and provide more stable and robust input representations for subsequent modeling.

#### 2.1.3. Data Preprocessing and Noise Reduction

To ensure reproducibility and consistent model inputs across all comparative experiments, a unified preprocessing and feature extraction pipeline was applied to the entire audio dataset. All recordings were resampled to 16 kHz and amplitude normalized. Subsequently, 80-dimensional log-Mel spectrograms were extracted using the short-time Fourier transform (STFT) with a frame length of 25 ms, a hop size of 10 ms, and a frequency range of 0–8 kHz. To reduce interference from non-informative segments, an energy-threshold-based method was used to remove leading and trailing silent frames. This shared input construction pipeline was consistently applied during the training, validation, and testing stages for all models.

Spectral gating was not implemented as an offline preprocessing step but was instead integrated as an adaptive input enhancement module within the ATF-Conformer framework. In brief, the module estimates a noise-suppression mask in the log-Mel domain and applies it before encoder input to suppress low-energy and noise-dominated time–frequency regions. The same gating strategy was used during both training and inference and was disabled in the ablation setting (“w/o spectral gating”) to evaluate its contribution. A formal description of the spectral gating mechanism is provided in Section 2.2.2, with full implementation details and hyperparameter settings given in Supplementary Section S1.

#### 2.1.4. Data Partitioning and Evaluation Protocols

To minimize information leakage arising from shared acoustic environments, microphone placement, and within-session correlations, all data splitting and evaluation procedures in this study followed a grouped mutual-exclusion principle, ensuring that samples from the same group were never assigned to both training and evaluation subsets.

A two-stage evaluation strategy was adopted to clearly separate model development from final assessment. First, a fixed group-wise held-out split with an intended 8:1:1 ratio was constructed. Under this grouped split, the full dataset was divided into 4739 development samples and 529 held-out test samples in Table 3. Within the development set, the grouped split corresponded approximately to 4202 training samples and 537 validation samples. Because the split was enforced at the group level rather than at the individual-sample level, the resulting sample counts were approximately, rather than exactly, in an 8:1:1 ratio. Class-wise distributions across the three subsets were kept broadly balanced, with 839/108/106 cough samples, 872/110/110 scream samples, 858/110/108 estrus samples, 821/105/103 feeding samples, and 812/104/102 normal samples assigned to the training, validation, and held-out test subsets, respectively.

The held-out test set was never used for model selection, hyperparameter tuning, or architectural design decisions. All ablation and robustness results reported on the held-out test set are strictly post hoc analyses conducted after the final model configuration was fixed.

All model comparisons and statistical analyses were conducted using five-fold grouped cross-validation on the development set. Performance metrics are reported as mean ± standard deviation across folds. Validation predictions from all folds were concatenated to form out-of-fold (OOF) outputs, which were used to construct confusion matrices such that each sample was evaluated exactly once by a model not trained on its corresponding group.

In addition, session-wise leave-one-session-out (LOSO) evaluation was performed to assess cross-session generalization. In this protocol, one recording session (morning, noon, or evening) was held out as the test domain, while the remaining sessions were used for training and validation under identical settings.

For routine group-housing recordings, groups were defined as g(x) = (pen_id, recording date, recording session), where the recording session denotes the time period within a day (morning, noon, or evening). This “pen × date × session” grouping was treated as an approximate independent unit to reduce correlation bias induced by shared background sound fields.

For targeted cough recordings collected from individual observation pens, an additional pig-wise constraint was applied based on pig identity, such that all directed cough segments from the same pig were assigned to a single subset or fold without overlapping. No automated pig identification algorithm was used in this study. For targeted close-range cough recordings, pig identity was confirmed by trained farm staff based on direct observation, routine husbandry records, and monitoring information during individual observation, and was used solely to enforce mutual exclusion during data partitioning rather than as a model input. Cough samples extracted from routine group-housing recordings followed the same session-level grouping strategy as other sound categories. This constraint explicitly prevents the model from exploiting individual-specific acoustic traits or recording-distance artifacts when learning cough-related patterns.

For inference-time additive noise robustness evaluation, background noise segments were sampled from a noise pool that was group-wise exclusive from the test samples, such that a noise segment n was selected only when g(n) ≠ g(x). This prevented shared session-level background characteristics between perturbed test samples and the added noise.

### 2.2. Method

#### 2.2.1. ATF-Conformer Framework

**Figure 4:**
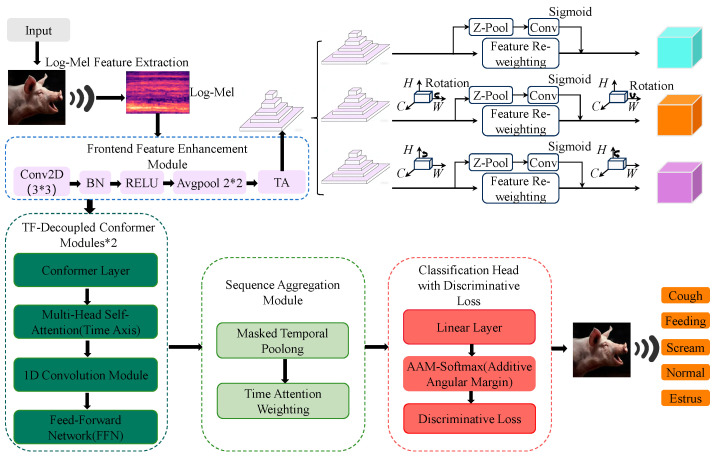
Architecture of the proposed ATF-Conformer model. The input audio is first converted into a log-Mel spectrogram and processed by a front-end enhancement module including spectral gating, Conv2D, batch normalization, ReLU, average pooling, and Triplet Attention. The resulting features are then encoded by the TFD-Conformer module, in which multi-head self-attention captures long-range temporal dependencies and a 1D convolution branch models local spectral patterns. After sequence aggregation through time attention weighting and masked temporal pooling, the utterance-level embedding is fed into a linear classification head trained with AAM-Softmax to predict five pig vocalization categories.

In real pig barn soundscapes, multi-class behavioral vocalization recognition is challenged by temporal non-stationarity, partial overlap across frequency bands, and behavior-dependent rhythmic variability [27]. To address these challenges, we propose ATF-Conformer, a unified sound-driven framework that integrates front-end enhancement, time–frequency decoupled sequence encoding, robust temporal aggregation, and discriminative classification, as shown in Figure 4. Triplet attention (TA) is employed to recalibrate salient acoustic cues across channel, temporal, and frequency dimensions [28], while the core encoder is derived from the Conformer architecture and redesigned with a time–frequency decoupled structure to improve robustness under noisy conditions [29].

In this study, the model takes the preprocessed log-Mel spectrogram X as input. The complete preprocessing pipeline and the spectral gating-based noise reduction strategy are described in the Supplementary Materials. The overall inference process can be summarized as

(1) $$\hat{y}=Cls\left(Pool\left(Enc\left(TA\left(Conv\left(X\right)\right)\right)\right)\right)$$

where Conv (.) denotes local feature extraction, TA (⋅) represents triplet attention, Enc (⋅) is the time–frequency decoupled Conformer encoder, Pool (⋅) denotes mask-based temporal pooling, and Cls (⋅) is trained using a discriminative objective.

Following the overall formulation in Equation (1), Figure 4 further illustrates how the input representation is processed through each functional block of ATF-Conformer. After front-end enhancement, the feature map is reshaped along the temporal axis into a frame sequence, where each time step corresponds to a pooled frequency-channel representation that is then fed to the TFD-Conformer encoder. The input audio is first represented as a log-Mel spectrogram. In the front-end enhancement module, spectral gating is used to attenuate noise-dominated time–frequency components, while the Conv2D–BN–ReLU–AvgPool stack extracts local acoustic patterns and reduces redundant variation. Triplet Attention (TA) then recalibrates the feature maps through three complementary branches, corresponding to channel–time, channel–frequency, and time–frequency interactions, so that salient behavior-related regions can be emphasized.

The enhanced features are then passed to the TFD-Conformer module. Within this module, the Conformer layer serves as the basic encoding block. Multi-head self-attention is responsible for modeling long-range temporal dependencies, which are important for rhythmic and sequential vocal patterns, whereas the 1D convolution module captures local spectral structure and short-range frequency-related patterns. The feed-forward network further projects and refines the fused representation. This design implements the intended time–frequency decoupling by assigning global modeling capacity to the temporal dimension and local modeling capacity to the frequency-related dimension.

Next, the sequence aggregation module converts frame-level representations into a fixed-length embedding. Time attention weighting assigns adaptive importance to different frames, and masked temporal pooling reduces the influence of silent or low-information regions. The resulting utterance-level embedding is finally passed to the classification head, which consists of a linear projection layer followed by AAM-Softmax. This classification strategy improves inter-class separation and supports discriminative learning for the five target pig vocalization categories. For reproducibility, the complete forward-pass procedure is summarized in the Supplementary Materials (Algorithm S1).

#### 2.2.2. Front-End Feature Enhancement Module

The front-end enhancement module aims to increase the density of behavior-discriminative information before sequence modeling while reducing interference from background components. Spectral gating is used to suppress noise-dominated time–frequency units. The full definition and hyperparameter settings are described in Supplementary Section S1. In the main model, this gating operation is applied before the Conv2D front end so that subsequent feature extraction is performed on adaptively denoised spectro-temporal inputs. The ablation setting “w/o Spectral Gating” bypasses this step while keeping all other components unchanged.

A lightweight 2D convolutional front end extracts local time–frequency patterns from the input spectrogram X and produces an intermediate representation

(2) $$H_{c}=Conv\left(X\right)\in R^{C^{\prime }\times F^{\prime }\times T^{\prime }}$$

where $X\in R^{F\times T}$ denotes the input log-Mel spectrogram, with F and T representing the numbers of frequency bins and time frames, respectively. $Conv\left(\cdot \right)$ denotes the front-end local feature extraction module implemented by the Conv2D–BN–ReLU–AvgPool stack. $H_{c}$ is the intermediate feature map produced by this module, and $H_{c}\in R^{C^{\prime }\times F^{\prime }\times T^{\prime }}$, where $C^{\prime }$ is the number of output channels, and $F^{\prime }$ and $T^{\prime }$ denote the transformed frequency and temporal dimensions after convolution and pooling operations.

This stage captures short-term and narrowband structures but may still retain redundant components under complex barn noise, motivating subsequent saliency recalibration.

Triplet Attention recalibrates features along three complementary views: channel–time (C–T), channel–frequency (C–F), and time–frequency (T–F) [28]. For each view $d\in \left\{CT,CF,TF\right\}$, an attention map $a_{d}\left(\cdot \right)$ is applied to reweight features:

(3) $$H_{d}=a_{d}\left(H_{c}\right)⊙H_{c},d\in \left\{CT,CF,TF\right\}$$

where ⊙ denotes element-wise multiplication. The final attention-enhanced feature is obtained by fusing the three branches:

(4) $$H_{TA}=\frac{1}{3}\left(H_{CT}+H_{CF}+H_{TF}\right)$$

This recalibration emphasizes behavior-relevant regions across channel/time/frequency and reduces spurious activations caused by non-stationary background interference.

#### 2.2.3. TF-Decoupled Conformer Encoder

Discriminative cues in pig vocalizations reside simultaneously in temporal rhythms and local spectral textures, whereas entangled time–frequency modeling can absorb incidental co-occurrences induced by non-stationary farm noise as class evidence, leading to spurious cross-dimensional correlations and increased confusion under acoustically overlapping conditions [30]. Compared with dual-path or axial-attention variants that impose global modeling along both axes [31], we adopt an asymmetric decoupling strategy that combines time-global attention with frequency-local convolution and fuses the two types of evidence in a higher-level semantic space.

Unlike dual-path architectures or axial-attention Transformers that perform symmetric global modeling along both time and frequency axes, our TFD-Conformer introduces an asymmetric inductive bias by explicitly assigning global modeling capacity to the temporal axis while restricting the frequency axis to local convolutional modeling. This design intentionally avoids learning spurious time–frequency co-occurrence patterns induced by non-stationary barn noise and forces the model to capture behavior-related temporal rhythms and frequency textures through two complementary but structurally separated branches.

Given the front-end enhanced feature $H_{TA}$, the temporal branch captures long-range dependencies via multi-head self-attention:

(5) $$H_{T}=MHSA\left(H_{TA}\right)$$

In implementation, the temporal branch treats the sequence length as the primary modeling axis, whereas the frequency branch applies local convolution over neighboring spectral bins to preserve short-range frequency structure without introducing full-axis global attention.

(6) $$H_{F}={Conv}_{1\times k}\left(H_{TA}\right)$$

The two representations are concatenated and projected into a unified sequence through a feed-forward network:

(7) $$H=FFN\left(\left[H_{T};H_{F}\right]\right)$$

where $\left[\cdot ;\cdot \right]$ denotes channel-wise concatenation. This “time-global, frequency-local” division of labor reduces coupling risks while preserving complementary evidence for robust multi-class recognition in complex barn soundscapes.

#### 2.2.4. Sequence Aggregation Module

Pig behavioral vocalizations often exhibit variable durations and temporal irregularity, and some frames may be silent or dominated by background interference, making uniform aggregation suboptimal [32]. To obtain compact and robust utterance-level representations, ATF-Conformer adopts mask-based temporal pooling to attenuate non-informative frames while emphasizing behavior-relevant segments.

Given the encoder output $H=\left[h_{1},\ldots ,h_{T}\right]\in R^{T\times D}$, where T denotes the number of frames, D is the feature dimension, and $h_{t}\in R^{D}$ is the representation of the t-th frame, temporal attention weights are computed as

(8) $$\alpha =softmax\left(W_{p}H\right)$$

where $W_{p}\in R^{1\times D}$ is a learnable projection vector, and $\alpha =\left[\alpha_{1},\ldots ,\alpha_{T}\right]\in R^{T}$ denotes the normalized importance weights assigned to individual frames.

To further suppress silent or low-information frames, a frame-level mask $m_{t}$ is introduced, where $m_{t}=0$ indicates a suppressed frame and $m_{t}=1$ indicates a valid frame. In this study, the mask was derived from frame-level energy activity after preprocessing and was used consistently during both training and inference to exclude silent or near-silent frames from temporal aggregation. The pooled utterance-level embedding is then obtained by

(9) $$z=\frac{\sum_{t=1}^{T}m_{t}\;\alpha_{t}\;h_{t}}{\sum_{t=1}^{T}m_{t}\;\alpha_{t}+\epsilon }$$

where $z\in R^{D}$ is the aggregated embedding, $\alpha_{t}$ is the temporal attention weight of frame t, and ϵ is a small constant added for numerical stability. In this way, the sequence aggregation module emphasizes behavior-relevant frames while reducing the influence of silent and noise-dominated regions.

#### 2.2.5. Classification Head with Discriminative Loss

After sequence aggregation, the embedding z is mapped to class logits via a linear classification head. To enhance inter-class separability under acoustic overlap, we train the model using Additive Angular Margin Softmax (AAM-Softmax). For sample i with ground-truth class $y_{I}$, the objective is defined as

(10) $$L=-\log\frac{\exp\left(\mathrm{scos}\left(\theta_{y_{i}}+m\right)\right)}{exp(scos(\theta_{y_{i}}+m))+\sum_{j\neq y_{i}}exp(scos(\theta_{j}))}$$

where $\cos\left(\theta_{j}\right)$ denotes the cosine similarity between embedding z and the class weight vector, m is the angular margin, and s is a scaling factor. The AAM-Softmax setting used in this work is reported in Supplementary Materials.

## 3. Results

### 3.1. Experimental Settings

#### 3.1.1. Baseline Models

To evaluate the effectiveness of ATF-Conformer for multi-class pig vocalization recognition under real farm conditions, we compared it with several representative baseline models commonly used in environmental audio and bioacoustics, including CRNN [33], PANNs [34], HTS-AT [35], the standard Conformer [29], and ECMISM [36]. These models span convolutional, recurrent, Transformer-based, and Conformer-family architectures, enabling a comprehensive comparison across major acoustic modeling paradigms. Specifically, CRNN was selected as a classical convolutional–recurrent baseline widely used in acoustic event recognition. PANNs was included as a representative CNN-based audio classification framework with strong feature extraction capabilities. HTS-AT was chosen to represent hierarchical Transformer-based audio modeling. The standard Conformer was used as the most direct backbone-level reference for our proposed encoder design. ECMISM was included as a stronger Conformer-family baseline with enhanced architectural components beyond the standard Conformer. Together, these baselines provide comparisons ranging from conventional acoustic sequence modeling to recent attention-based and Conformer-based architectures. In this way, the benchmark set covers both lightweight conventional models and more recent high-capacity attention-based encoders, enabling a more balanced evaluation of robustness and discriminative performance under realistic farm acoustic conditions.

All models were trained from scratch using identical data partitions, input features, and training protocols to ensure a fair comparison. The network architecture and key parameter settings are shown in Table 4.

Compared with these baselines, ATF-Conformer introduces front-end spectrogram enhancement, multidimensional interactive attention, and a time–frequency decoupled Conformer encoder, with the goal of improving robustness to non-stationary farm noise and overlapping vocalization patterns.

#### 3.1.2. Experimental Environment and Hyperparameters

All models were trained using the PyTorch 2.1.0 (Meta AI, Menlo Park, CA, USA) framework on an NVIDIA A100 GPU (NVIDIA Corporation, Santa Clara, CA, USA). The Adam optimizer was employed with an initial learning rate of 0.001, along with learning rate scheduling and early stopping. Model selection was based on validation Macro-F1, and the checkpoint with the best validation performance within each training run was retained for subsequent evaluation. The batch size was set to 16, and the maximum number of training epochs was 100. To ensure reproducibility, a fixed random seed (seed = 42) was used for all experiments.

To ensure fair comparison, all models were retrained using identical data partitions, preprocessing pipelines, and feature configurations, including a sampling rate of 16 kHz, 80-dimensional log-Mel spectrograms, and the same normalization and silence trimming procedures. No external data or pretrained weights were used. Although PANNs and HTS-AT are often pretrained on large-scale datasets such as AudioSet in their original implementations, only their architectural designs were adopted in this study. Consequently, the reported results reflect architectural robustness under a controlled, no-pretraining setting rather than performance optimized through external data.

#### 3.1.3. Evaluation Metrics

1. Accuracy

Accuracy measures the overall correctness of the model’s predictions. It is defined as the proportion of correctly predicted samples, where a sample is considered correctly predicted if its predicted label exactly matches the true label. Mathematically, it is calculated as:

(11) $$\mathrm{Accuracy}=\frac{1}{N}\sum_{i=1}^{N}I\left({\hat{y}}_{i}=y_{i}\right)$$

here, N represents the total number of samples, and $I\left(\cdot \right)$ is the indicator function, which equals 1 if the predicted label ${\hat{y}}_{i}$ matches the true label $y_{i}$, and 0 otherwise.

2. Precision

Precision measures the accuracy of positive predictions. For each class, it calculates the proportion of predicted positive samples that belong to that class, reflecting how well the model avoids false positives. It is calculated as:

(12) $${\mathrm{Precision}}_{\mathrm{macro}}=\frac{1}{C}\sum_{i=1}^{C}\frac{TP_{i}}{TP_{i}+FN_{i}}$$

where C is the number of classes, $TP_{i}$ represents true positives (samples that are correctly predicted as class i), and $FP_{i}$ represents false positives (samples predicted as class i but belonging to another class).

3. Recall

Recall, also known as Sensitivity or True Positive Rate, measures how well the model can identify all the true instances of a given class. It is defined as the proportion of true positive samples that are correctly identified by the model, reflecting the model’s ability to avoid false negatives. Recall is calculated as:

(13) $${\mathrm{Recall}}_{\mathrm{macro}}=\frac{1}{C}\sum_{i=1}^{C}\frac{TP_{i}}{TP_{i}+FN_{i}}$$

here, $FN_{i}$ represents false negatives (samples that belong to class i but were misclassified).

4. F1-score

The F1-score is the harmonic mean of Precision and Recall, providing a single metric that balances the trade-off between false positives and false negatives. It is particularly useful when the class distribution is imbalanced. F1-score is computed as:

(14) $${F1}_{macro}=\frac{1}{C}\sum_{i=1}^{C}\frac{2{Precision}_{i}{Recall}_{i}}{{Precision}_{i}{+Recall}_{i}}$$

This metric combines both Precision and Recall into one value, ensuring that both false positives and false negatives are considered in the evaluation.

5. AUROC

The AUROC (Area Under the Receiver Operating Characteristic Curve) evaluates the model’s ability to distinguish between different classes without being sensitive to class imbalance. It is calculated using a one-vs-rest strategy, where a binary classification is performed for each class, and the area under the ROC curve is computed for each class. Macro-AUROC is the average of the AUROCs across all classes:

(15) $$\mathrm{Macro}\text{-}\mathrm{AUROC}=\frac{1}{C}\sum_{i=1}^{C}{\mathrm{AUROC}}_{i}$$

This metric reflects the threshold-independent discriminative ability of the model across all classes, with higher values indicating better performance in distinguishing between classes.

6. CM

The CM (Confusion Matrix) is a diagnostic tool that visualizes the performance of a classification model by showing the true versus predicted labels for each class. It quantifies the number of misclassifications and correct classifications, enabling the identification of major confusion pairs and error patterns. The confusion matrix is defined as:

(16) $$\mathrm{CM}=\left[CM_{i,j}\right],\;\;\;\;\;i,j=1,\ldots ,C$$

here, ${\mathrm{CM}}_{i,j}$ represents the number of samples whose true class is i and were predicted as class j. The confusion matrix helps to analyze the relationships between different classes, providing insights into which pairs of classes are often confused by the model.

Together, these complementary metrics were used to provide a balanced assessment of overall accuracy, class-wise discrimination, threshold-independent separability, and error structure under grouped evaluation.

### 3.2. Analysis of Training Accuracy and Loss

**Figure 5:**
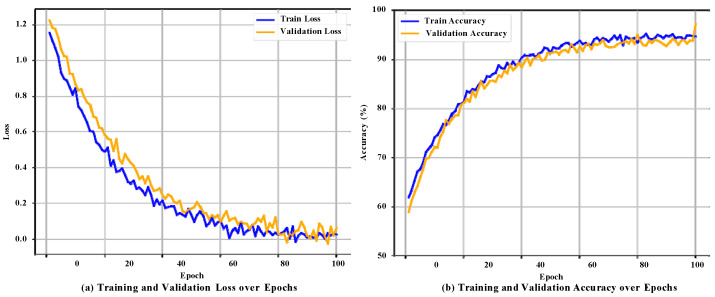
Training dynamics of ATF-Conformer over 100 epochs. (**a**) Training and validation loss; (**b**) Training and validation accuracy.

Figure 5 illustrates the training dynamics of ATF-Conformer under the fixed grouped split and is provided mainly to show optimization stability and convergence behavior.

Figure 5a shows that the model converges quickly in the first 30 epochs, with both training and validation losses decreasing sharply from around 1.2 to below 0.3, indicating effective learning of discriminative acoustic features. The loss stabilizes after around the 80th epoch, with the training loss remaining at approximately 0.05 and validation loss at 0.08, demonstrating stable optimization with no overfitting.

Figure 5b shows that validation accuracy reaches 97.46% at epoch 100, with comparable performance observed on the held-out test set (97.38%). These results, obtained from a single fixed 8:1:1 split, illustrate typical training dynamics, while final performance conclusions are based on more robust statistics from 5-fold grouped cross-validation. The small discrepancy between training and validation accuracy suggests stable optimization under the fixed split, whereas final performance conclusions are based on the grouped cross-validation and held-out evaluations reported below.

### 3.3. Ablation Studies

Following the held-out evaluation protocol described in Section 2.1.4, ablation experiments were conducted to quantify the contribution of each key component of ATF-Conformer under complex farm noise conditions. Each module was removed individually while the remaining architecture and hyperparameters were kept fixed. Performance was evaluated on the held-out test set after the final model and hyperparameters were fixed, using Accuracy, Macro-Precision, Macro-Recall, and Macro-F1 as summarized in Table 5.

As shown in Table 5, the full ATF-Conformer achieves the best overall performance, and removing any individual component leads to a noticeable degradation, indicating that all modules contribute positively to the final model.

Specifically, removing spectral gating or Triplet Attention (TA) leads to comparable performance drops, highlighting their respective roles in suppressing background noise and selectively enhancing informative time–frequency regions. Eliminating the TFD-Conformer results in the most substantial degradation, underscoring the importance of complementary temporal and spectral modeling enabled by the time–frequency decoupled design, particularly for capturing long-range temporal dependencies and stable spectral patterns when distinguishing acoustically similar behaviors such as scream and estrus. When only the convolutional front end is retained, performance degrades markedly, indicating that local convolutional features alone are insufficient for robust behavior recognition in noisy farm environments.

### 3.4. Performance Comparison with Baseline Models

Table 6 presents the main comparative results under the unified 5-fold grouped cross-validation protocol and shows that ATF-Conformer achieves the best overall performance across the reported metrics. In particular, ATF-Conformer yields the highest Accuracy, Macro-F1, and Macro-AUROC with the smallest or near-smallest variability across folds.

ATF-Conformer achieves consistently strong performance across all major metrics with the smallest variance across folds, reflecting high accuracy and stable cross-group generalization. Relative to ECMISM, the strongest baseline, ATF-Conformer shows improved Accuracy and Macro-F1 under identical evaluation settings. It also exhibits a more balanced precision–recall trade-off, whereas several baseline models demonstrate asymmetric behavior, including higher precision but reduced recall. In contrast, convolutional and recurrent architectures show lower Macro-F1 scores and greater variability, indicating increased sensitivity to background noise and group-wise domain shifts. ATF-Conformer further attains the highest Macro-AUROC, suggesting stronger threshold-independent discrimination.

### 3.5. Error Structure and Confusion Analysis

**Figure 6:**
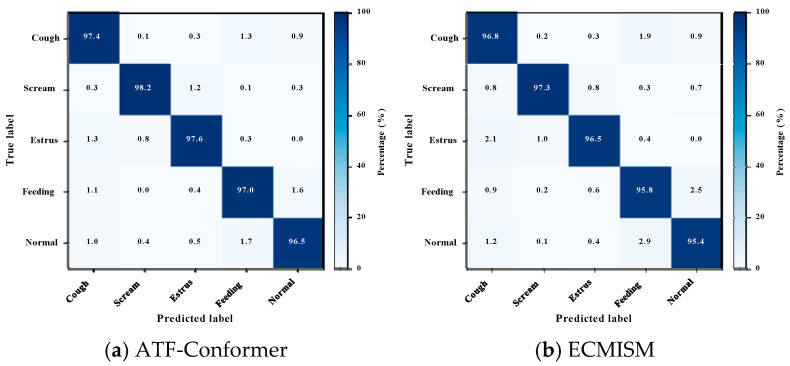

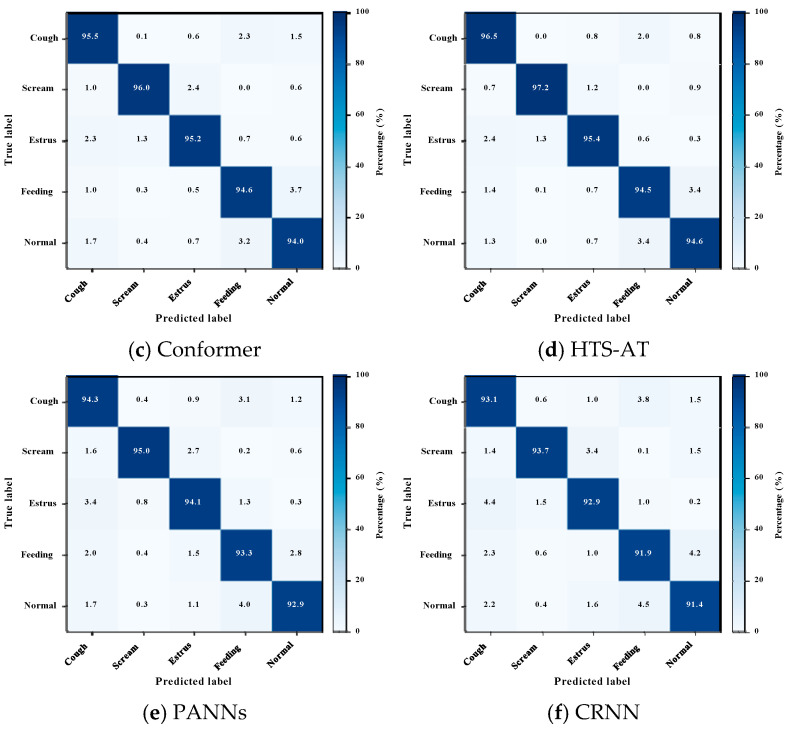
Row-normalized confusion matrices of (**a**) ATF-Conformer, (**b**) ECMISM, (**c**) Conformer, (**d**) HTS-AT, (**e**) PANNs, and (**f**) CRNN based on out-of-fold predictions under 5-fold grouped cross-validation. Rows denote ground-truth labels and columns denote predicted labels. Values are row-normalized percentages and rounded to one decimal place.

Figure 6 further clarifies the error structure behind the aggregate metrics by showing the confusion patterns of different models under acoustically similar or overlapping conditions.

Row-normalized confusion matrices are constructed from out-of-fold (OOF) predictions obtained under 5-fold grouped cross-validation, where each sample is predicted exactly once by a model trained on mutually exclusive groups. Aggregating predictions across all folds enables a robust characterization of error structures without involving any information from the held-out test set. The resulting confusion matrices for all models are shown in Figure 6.

Across all models, misclassifications are primarily concentrated among similar acoustic low-energy classes, with mutual confusion between Feeding and Normal being the most prominent. Occasional confusion involving the weak-energy, rhythmical Estrus class is also observed. These patterns are consistent with real pig farm soundscapes, where substantial time–frequency overlap and persistent low-frequency background noise are common.

Compared with baseline models, ATF-Conformer exhibits more balanced diagonal dominance across all five behavior categories and shows a clear suppression of dominant confusion pairs. Cross-class triggering between high-energy events and weak-energy categories is also better controlled, indicating improved preservation of class-discriminative cues under background similarity and frequency-band overlap.

In contrast, transformer-based baselines such as Conformer and HTS-AT show higher mutual confusion between Feeding and Normal, while convolutional and recurrent models (PANNs and CRNN) exhibit more pronounced error propagation across low-energy and rhythmical classes. These results indicate greater susceptibility to background interference and reduced stability under complex acoustic conditions.

Although residual confusion between Feeding and Normal remains, ATF-Conformer reduces this error more effectively than all baseline models.

### 3.6. Per-Class Performance Analysis

Table 7 summarizes the per-class classification results for the five pig behavioral vocalizations. Overall, ATF-Conformer maintains consistently strong performance across all five classes, with particularly clear advantages for weak-energy and acoustically overlapping categories.

(1) Cough: ATF-Conformer achieves the highest F1-score and AUROC for cough, indicating strong sensitivity to brief respiratory events under noisy barn conditions. This result suggests improved discrimination of short transient cues relative to the baseline models.
(2) Scream: For scream, ATF-Conformer achieves performance comparable to or better than the strongest baseline models and attains the highest AUROC. This indicates stable recognition of high-energy abnormal vocalizations across decision thresholds.
(3) Estrus: ATF-Conformer shows the best overall performance for estrus recognition. The improvement is particularly relevant for this relatively weak-energy and rhythmical class under complex farm acoustic conditions.
(4) Feeding: For feeding sounds, ATF-Conformer achieves the highest overall F1-score and AUROC while maintaining a balanced precision–recall trade-off. This suggests improved robustness for a category that overlaps strongly with low-frequency barn background activity.
(5) Normal: ATF-Conformer also performs best overall for the normal class, with the highest F1-score and AUROC. This result indicates more stable discrimination between routine barn acoustics and behavior-related vocal events.

Taken together, these results show that ATF-Conformer achieves consistently strong and well-balanced per-class performance across the five pig vocalization categories.

### 3.7. Robustness and Cross-Session Generalization

#### 3.7.1. Robustness Evaluation Under Additive Noise Perturbations

To evaluate robustness against additional background noise during inference, additive noise perturbation experiments were conducted on the held-out test set without retraining or threshold recalibration. Real pig farm background noises, including ventilation, feeding equipment, pen friction, and human activities, were extracted from non-event segments and mixed with test samples at multiple signal-to-noise ratio (SNR) ranges, following the same feature extraction pipeline as the main experiments. This evaluation reflects performance degradation caused purely by noise interference.

**Figure 7:**
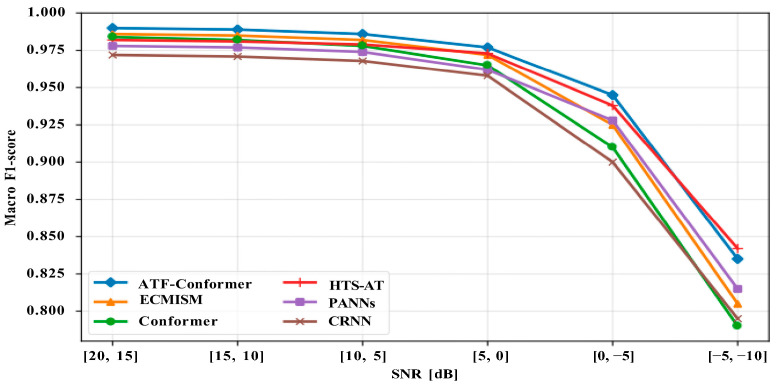
Macro-F1 as a function of SNR range under additive noise perturbations on the held-out test set for ATF-Conformer and baseline models (ECMISM, Conformer, HTS-AT, PANNs, and CRNN).

Figure 7 shows the Macro-F1 degradation trends across different SNR ranges. As SNR decreases, all models exhibit a gradual performance decline, with degradation accelerating below 0 dB, where strong noise significantly increases class overlap and boundary ambiguity. At higher SNR levels (≥5 dB), performance differences among models remain relatively small.

Across most SNR ranges, ATF-Conformer consistently maintains higher Macro-F1 scores and a more gradual degradation trend, indicating stronger robustness to noise perturbations. HTS-AT remains competitive at extremely low SNR levels, while PANNs, CRNN, and the standard Conformer experience substantially larger performance drops, suggesting higher sensitivity to severe noise conditions.

#### 3.7.2. Cross-Session Generalization Experiment

Cross-session generalization was evaluated using a session-wise leave-one-session-out (LOSO) protocol across three recording periods (Morning, Noon, and Evening). In each run, one session was held out for testing, while the remaining two sessions were used for training and validation under the same configuration as the main experiments. To reduce information leakage caused by shared backgrounds, the training–validation split was performed only within the non-test sessions. The held-out session was not involved in model selection or hyperparameter tuning. Macro-F1 was used as an evaluation metric. Table 8 summarizes the Macro-F1 scores on the three held-out sessions and the cross-session mean ± SD.

Since using Noon as the test domain results in a more consistent performance degradation across models, a Noon degradation metric (ΔNoon) is defined to quantify performance loss due to this domain shift:

(17) $$\Delta Noon=\left(\frac{Morning+Evening}{2}\right)-Noon.$$

Here, Morning, Noon, and Evening denote the Macro-F1 (%) obtained when the corresponding session is used as the held-out test domain. A smaller ΔNoon indicates lower sensitivity to the Noon domain shift and stronger cross-session robustness.

**Figure 8:**
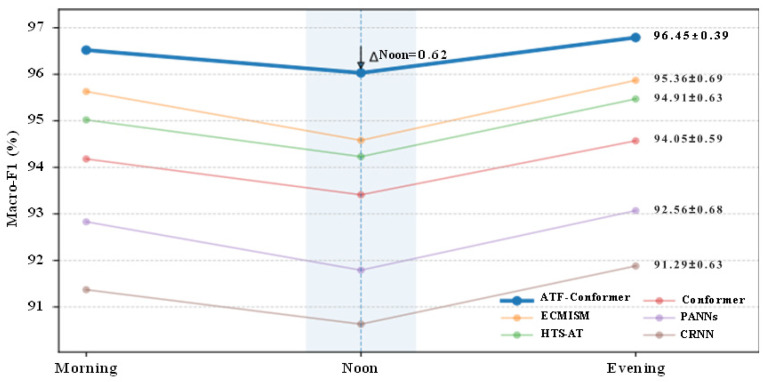
Session-wise cross-session generalization results of different models under the LOSO protocol, reported as Macro-F1 (%) on Morning, Noon, and Evening held-out tests.

As shown in Figure 8, all models experience the largest performance drop when Noon is used as the test domain, likely due to increased acoustic variability during midday (e.g., feeding frequency changes, human activity, and equipment operation), which amplifies background disturbances and class overlap. ATF-Conformer achieves the highest or jointly highest Macro-F1 across all three sessions and exhibits the smallest cross-session variability (96.45 ± 0.39). More importantly, it attains the lowest ΔNoon value (0.62), substantially smaller than those of ECMISM (1.17), HTS-AT (1.02), and Conformer (0.97), indicating reduced sensitivity to the Noon domain shift and stronger cross-session generalization.

## 4. Discussion

### 4.1. Behavioral Vocalization Characteristics and Error Patterns in Commercial Pig Barns

This study shows that ATF-Conformer can recognize five categories of pig behavioral vocalizations under complex soundscape conditions in a real large-scale pig farm. Consistent performance across multiple evaluation settings suggests that the model has potential for practical use in acoustic monitoring under similar commercial conditions.

The dominant confusion patterns observed can be largely attributed to inherent acoustic similarity among low-frequency behaviors and the characteristics of real barn soundscapes. Feeding and normal behavior sounds share long-duration low-frequency components accompanied by activity-related background noise, such as chewing friction, pen contact, and ventilation hum. As a result, their discriminability relies more on subtle rhythmic regularities and fine-grained spectral texture differences rather than coarse energy magnitude, making the class boundary particularly sensitive to background variability and overlapping sound sources.

Estrus vocalizations, although rhythmical, are typically weak in energy and therefore more susceptible to partial masking or temporal fragmentation under multi-source interference. Once rhythmic consistency is disrupted, the remaining spectral fragments may resemble other vocalization types, leading to occasional cross-class triggering. These error patterns reflect intrinsic challenges of real pig barn soundscapes rather than simple model deficiencies.

### 4.2. Robustness of Time–Frequency Modeling Under Noisy Farm Conditions

The effectiveness of the proposed framework is largely driven by the time–frequency decoupled encoding strategy. This design introduces an inductive bias that emphasizes long-range temporal modeling, which is particularly informative for pig vocalizations with rhythmic or repetitive structures. By constraining frequency modeling to local receptive fields while preserving global temporal dependencies, ATF-Conformer mitigates noise-driven time–frequency entanglement and stabilizes decision boundaries under non-stationary barn noise. Spectral gating and Triplet Attention further enhance robustness by suppressing noise-dominated regions and emphasizing salient time–frequency patterns, forming a progressive pipeline of input purification and saliency recalibration [37]. Mask-based temporal pooling and AAM-Softmax additionally improve separability among acoustically similar behavior categories.

### 4.3. Implications for Practical Deployment in Precision Livestock Farming

From an application perspective, segment-level predictions can be integrated over time to support practical pen-level screening and early-warning management in commercial farms. Repeated cough detections within the same pen or monitoring window can be used to flag animals or pens for targeted inspection, thereby helping farm owners or workers identify suspected respiratory cases for further on-site assessment. Recall-prioritized thresholding may therefore be applied to health-related cough events to reduce missed detections [38], while stricter thresholds can be used for high-energy abnormal events such as screams to suppress false alarms. Persistently confusable categories such as feeding and normal behavior sounds are better treated as indicators of group-level state trends rather than direct alarm triggers. Compared with multimodal approaches, the proposed unimodal acoustic framework reduces system complexity and deployment cost, which is advantageous for large-scale farm environments [39]. In addition, the unimodal framework is particularly beneficial for applications where fine-grained recognition of individual behaviors is required, as seen in the recognition of sow nursing behavior [40].

### 4.4. Limitations and Future Perspectives

Robustness and cross-session generalization analyses further support practical applicability. Models relying on patch- or token-based global attention, such as HTS-AT and Swin-style architectures, may amplify background-dependent correlations in noisy barns. In contrast, ATF-Conformer exhibits more gradual performance degradation under additive noise and shows lower sensitivity to session-level domain shifts, indicating more stable generalization [41].

Despite these advantages, external generalization remains a limitation of this study. Data were collected primarily from a single commercial farm, and transferability across different farms, seasons, recording devices, and microphone–animal distances requires further validation. Although pig-wise mutual exclusion and group-wise splitting were applied to mitigate individual- and session-level bias, targeted close-range cough acquisition may still introduce residual distance- or setup-related effects. Future work will therefore focus on improving generalization through cross-farm validation with heterogeneous recording configurations, expanding multi-center and multi-season data collection, extending the framework from short-segment classification to continuous event detection, and exploring self-supervised learning and lightweight multimodal fusion to enhance robustness under complex environmental conditions [42].

## 5. Conclusions

In this study, we investigated acoustic recognition of five pig behavioral vocalizations under real large-scale commercial farming conditions. By constructing an in situ vocalization dataset and applying the attention-guided time–frequency decoupled framework ATF-Conformer, we achieved stable classification performance across multiple behavior categories under strict group-wise evaluation. The results suggest that ATF-Conformer is robust under complex and non-stationary barn soundscape conditions and has potential for practical use in acoustic monitoring under similar commercial conditions. In particular, the proposed framework may support pen-level acoustic screening of frequent cough or abnormal vocal events and may assist farm owners or workers in prioritizing targeted on-site inspection after confirmation. Although the present study was limited to data collected from a single farm, these findings provide a methodological reference for acoustic-based pig behavior monitoring and for future developments in precision livestock farming systems.

## Figures and Tables

**Table 1:** Statistics of the pig behavioral sound dataset collected from a real pig farm.

Behavior Category	Sample Count	Average Duration (Seconds)	Frequency Range (kHz)
Cough	1053	1.82	0.8–3.0
Scream	1092	1.75	3.0–8.0
Estrus	1076	1.94	1.0–3.5
Feeding	1029	1.88	0.2–2.5
Normal	1018	1.80	0–1.5

Note: Sample count, mean duration, and typical dominant frequency band range. Frequency range indicates the dominant energy band estimated from class-wise averaged log-Mel spectrograms (see Supplementary Section S1 for details).

**Table 2:** Recording source and partition design by vocalization class.

Vocalization Class	Routine Source	Supplementary Source	Close-Range Sampling	Routine Split Unit	Additional Split Rule
Feeding	Finishing pens	–	–	Pen × date × session	–
Normal	Finishing pens	–	–	Pen × date × session	–
Scream	Finishing pens	–	–	Pen × date × session	–
Estrus	Breeding stalls	–	–	Session-level mutual exclusion	–
Cough	Finishing pens	Symptomatic pigs	Yes	Pen × date × session	Pig-wise exclusion *

Note: * Pig-wise exclusion indicates that, for targeted close-range cough recordings, samples from the same pig were assigned to only one subset. For routine group-housing recordings, groups were defined by pen ID × recording date × recording session (morning, noon, or evening).

**Table 3:** Class-wise sample distribution across the training, validation, and held-out test subsets under the grouped 8:1:1 partition.

Vocalization Class	Train	Validation	Test	Total
Cough	839	108	106	1053
Scream	872	110	110	1092
Estrus	858	110	108	1076
Feeding	821	105	103	1029
Normal	812	104	102	1018
Total	4202	537	529	5268

Note: The split was implemented at the group level rather than at the individual-sample level. Therefore, the final sample counts are approximately, rather than exactly, in an 8:1:1 ratio. For routine group-housing recordings, each group was defined by pen ID × recording date × recording session, whereas targeted close-range cough samples were additionally constrained by pig-wise mutual exclusion.

**Table 4:** Main architectural components and key settings of ATF-Conformer and baseline models used for five-class pig vocalization classification.

Network	Component	Configuration/Value
ATF-Conformer	Front-end encoding	3 × 3 2D Conv + BN + ReLU; 2 × 2 AvgPool
	Attention module	Triplet Attention
	Core encoder	TFD-Conformer × 2
	Aggregation/classifier	Masked temporal pooling + AAM-Softmax
ECMISM	Convolutional subsampling	Two-layer downsampling convolution
	Core encoder	Conformer × 2
	Auxiliary design	Skip fusion + InLoss
	Output strategy	Global pooling + 5-class output
Conformer	Convolutional subsampling	Two-layer 2D Conv
	Projection	Linear projection
	Core encoder	Conformer × 2
	Aggregation	Global average pooling
HTS-AT	Patch embedding	4 × 4 Conv, stride 4 × 4
	Hierarchical encoder	Swin Transformer (2/2/6/2)
	Token processing	Patch merging + token-semantic CNN
	Aggregation	Global average pooling
PANNs	Convolutional extraction	Stacked 3 × 3 Conv (64 → 128 → 256 → 512)
	Pooling	Progressive 2 × 2 average pooling
	Aggregation	GAP + GMP
	Classifier	FC 2048
CRNN	Convolutional extraction	3 × 3 Conv (64 → 128)
	Pooling	MaxPool (2 × 2, 3 × 3)
	Temporal modeling	LSTM (64)
	Classifier	FC 128

**Table 5:** Ablation results on the held-out test set (%).

Model	Accuracy (%)	Precision (%)	Recall (%)	F1-Score (%)
Full Model	97.38	97.86	97.45	97.65
w/o Spectral Gating	94.53	94.81	94.18	94.49
w/o TA	94.76	95.09	94.32	94.70
w/o TFD-Conformer	92.08	92.67	91.83	92.24
Conv-Front Only	88.57	89.29	88.04	88.62

Note: All results are evaluated on the held-out test set under the same protocol. Precision, Recall, and F1-score are macro-averaged across the five classes. No model selection, hyperparameter tuning, or architectural adjustment was performed based on the test set. Ablation results are reported strictly for post hoc analysis after model development was completed. w/o Spectral Gating denotes bypassing the input enhancement step in the ATF front end while keeping the shared feature extraction pipeline unchanged.

**Table 6:** Performance comparison of different models using 5-fold grouped cross-validation (mean ± SD).

Model	Accuracy ± SD (%)	Macro-Precision ± SD (%)	Macro-Recall ± SD (%)	Macro-F1 ± SD (%)	Macro-AUROC ± SD	*p*-Value vs. ATF
ATF-Conformer	97.34 ± 0.42	97.82 ± 0.23	97.41 ± 0.39	97.61 ± 0.28	0.97 ± 0.01	–
ECMISM	96.38 ± 0.53	97.01 ± 0.38	96.12 ± 0.58	96.52 ± 0.47	0.96 ± 0.01	0.008
Conformer	95.07 ± 0.72	95.42 ± 0.68	95.31 ± 0.71	95.39 ± 0.69	0.95 ± 0.01	<0.001
HTS-AT	95.66 ± 0.61	97.21 ± 0.47	94.23 ± 0.82	95.68 ± 0.59	0.95 ± 0.01	<0.001
PANNs	93.95 ± 0.79	93.34 ± 0.88	94.62 ± 0.81	94.02 ± 0.77	0.95 ± 0.01	<0.001
CRNN	92.61 ± 0.98	93.83 ± 0.92	91.94 ± 1.08	92.79 ± 0.96	0.93 ± 0.01	<0.001

Note: Metrics are computed on the validation set of each fold and reported as mean ± SD across the 5 folds. All metrics are reported in %, except AUROC (0–1). Macro-AUROC is computed from predicted probabilities in an OvR (one-vs-rest) manner and macro-averaged across classes. *p*-values are computed using a two-sided Wilcoxon signed-rank test on fold-wise Macro-F1 scores against ATF-Conformer. The Wilcoxon test is used due to the paired nature of fold-wise scores and the small sample size (n = 5 folds).

**Table 7:** Per-class performance comparison of ATF-Conformer and baseline models for the five pig vocalization categories (mean ± SD).

Vocalization Class	Model	Precision ± SD (%)	Recall ± SD (%)	F1-Score ± SD (%)	AUROC ± SD
Cough	ATF-Conformer	97.52 ± 0.83	98.21 ± 0.74	97.86 ± 0.72	0.98 ± 0.01
	ECMISM	97.08 ± 0.54	96.48 ± 0.62	96.78 ± 0.56	0.97 ± 0.01
	Conformer	95.94 ± 0.72	95.36 ± 0.81	95.65 ± 0.74	0.96 ± 0.02
	HTS-AT	96.41 ± 0.69	94.32 ± 0.88	95.35 ± 0.80	0.96 ± 0.02
	PANNs	93.26 ± 0.91	94.74 ± 0.96	93.99 ± 0.90	0.95 ± 0.02
	CRNN	92.96 ± 0.98	91.42 ± 1.07	92.18 ± 0.99	0.94 ± 0.03
Scream	ATF-Conformer	98.34 ± 0.67	97.48 ± 0.82	97.91 ± 0.69	0.97 ± 0.02
	ECMISM	97.62 ± 0.61	96.21 ± 0.71	96.91 ± 0.63	0.96 ± 0.02
	Conformer	96.26 ± 0.74	95.18 ± 0.83	95.72 ± 0.75	0.95 ± 0.02
	HTS-AT	99.12 ± 0.53	96.87 ± 0.79	97.97 ± 0.62	0.96 ± 0.02
	PANNs	93.47 ± 0.89	95.63 ± 0.91	94.54 ± 0.83	0.94 ± 0.03
	CRNN	93.52 ± 0.93	92.34 ± 1.02	92.92 ± 0.95	0.93 ± 0.03
Estrus	ATF-Conformer	96.78 ± 1.12	94.47 ± 1.06	95.63 ± 0.94	0.96 ± 0.02
	ECMISM	95.92 ± 0.76	93.62 ± 0.84	94.76 ± 0.75	0.95 ± 0.02
	Conformer	93.86 ± 0.83	94.08 ± 0.92	93.97 ± 0.84	0.94 ± 0.02
	HTS-AT	94.18 ± 0.92	92.51 ± 1.01	93.33 ± 0.91	0.94 ± 0.02
	PANNs	90.74 ± 1.09	93.36 ± 1.02	91.92 ± 1.01	0.93 ± 0.03
	CRNN	93.41 ± 1.02	91.96 ± 1.09	92.64 ± 1.01	0.93 ± 0.03
Feeding	ATF-Conformer	96.53 ± 0.88	95.81 ± 0.97	96.16 ± 0.91	0.96 ± 0.01
	ECMISM	96.18 ± 0.74	95.46 ± 0.83	95.82 ± 0.75	0.95 ± 0.02
	Conformer	94.64 ± 0.81	94.92 ± 0.88	94.78 ± 0.82	0.94 ± 0.02
	HTS-AT	94.82 ± 0.93	92.94 ± 1.01	93.87 ± 0.92	0.93 ± 0.02
	PANNs	94.06 ± 0.92	96.02 ± 0.91	95.03 ± 0.84	0.95 ± 0.02
	CRNN	93.26 ± 1.01	92.18 ± 1.08	92.71 ± 1.02	0.92 ± 0.03
Normal	ATF-Conformer	97.69 ± 0.54	98.97 ± 0.43	98.33 ± 0.36	0.99 ± 0.01
	ECMISM	97.84 ± 0.52	98.12 ± 0.51	97.98 ± 0.45	0.98 ± 0.01
	Conformer	95.62 ± 0.73	96.18 ± 0.71	95.90 ± 0.64	0.96 ± 0.02
	HTS-AT	95.46 ± 0.84	94.78 ± 0.92	95.12 ± 0.76	0.96 ± 0.02
	PANNs	96.68 ± 0.74	95.92 ± 0.83	96.30 ± 0.72	0.96 ± 0.01
	CRNN	94.52 ± 0.94	93.68 ± 1.01	94.09 ± 0.93	0.95 ± 0.03

**Table 8:** Cross-session generalization across recording sessions (Morning/Noon/Evening) evaluated by Macro-F1 (%) on session-wise held-out tests.

Model	Test Morning	Test Noon	Test Evening	Mean ± SD	ΔNoon
ATF-Conformer	96.52	96.03	96.79	96.45 ± 0.39	0.62
ECMISM	95.63	94.58	95.87	95.36 ± 0.69	1.17
HTS-AT	95.02	94.23	95.47	94.91 ± 0.63	1.02
Conformer	94.18	93.41	94.57	94.05 ± 0.59	0.97
PANNs	92.83	91.79	93.07	92.56 ± 0.68	1.16
CRNN	91.37	90.63	91.88	91.29 ± 0.63	1.00

Note: Mean ± SD is computed across the three held-out sessions (n = 3).

## Data Availability

The dataset generated and analyzed during this study is publicly available in the Zenodo repository (https://sandbox.zenodo.org/records/485328 (accessed on 9 April 2026)). The implementation code for the proposed ATF-Conformer framework is publicly available on GitHub (https://github.com/wangjianpinghist/ATF-Conformer (accessed on 20 April 2026)). Both the dataset and code are openly accessible to support reproducibility and further research.

## Supplementary Materials

The following supporting information can be downloaded at: https://www.mdpi.com/article/10.3390/ani16091337/s1. Table S1. Environmental parameters of the pig farm; Section S1. Data Preprocessing and Spectral Gating Noise Reduction; Figure S1. Audio feature extraction and preprocessing pipeline; Algorithm S1. Forward propagation of ATF-Conformer.

## Abbreviations

AAM-Softmax	Additive Angular Margin Softmax
ATF-Conformer	Attention-Guided Time-Frequency Decoupled Conformer
AUROC	Area Under the Receiver Operating Characteristic Curve
CM	Confusion Matrix
Log-Mel	logarithmic Mel-frequency
LOSO	Leave-one-session-out
OOF	out-of-fold
OvR	one-vs-rest
STFT	short-time Fourier transform
TA	Triplet Attention
TF	Time-Frequency
TFD-Conformer	Time-Frequency Decoupled Conformer
