# Supplementary material for: A Conformer-Based Time–Frequency Decoupling Network for Pig Vocalization Behavior Classification
Source: Animals (Basel). 2026 Apr 27;16(9):1337. doi: 10.3390/ani16091337 (PMC13162907; doi:10.3390/ani16091337)
Supplement: Supplementary file 1 [file animals-16-01337-s001.zip › animals-4276088-supplementary.pdf]

Supplementary Materials

Table S1. Environmental parameters of the pig farm.

Parameter	Value
Air temperature	18–24 °C
Relative humidity	50–70 %
Temperature–humidity index	70–75
Ambient background noise level	60–70 dB
Air velocity (ventilation)	0.1–0.5 m/s
Air exchange rate	8–12 times/hour
CO_2 concentration	< 1000 ppm
Ammonia (NH_3) concentration	< 25 ppm
Hydrogen sulfide (H_2S) concentration	< 10 ppm
PM2.5 / PM10 concentration	< 50 μg/m^3
Feeding time	1–2 hours/day
Cleaning and disinfection time	1–2 hours/day
Audio sampling frequency	16 kHz

Section S1. Data Preprocessing and Spectral Gating Noise Reduction

In real pig-farm soundscapes, raw audio is affected by non-stationary background noise, irregular behavior timing, and acoustic-condition variability. To obtain stable and discriminative inputs, we adopt a unified preprocessing pipeline consisting of log-Mel feature extraction, silence trimming, and a spectral gating strategy designed for classification-oriented noise suppression.

S1.1 Log-Mel extraction and silence trimming

Audio is processed at a sampling rate of 16 kHz. Log-Mel spectrograms are extracted using STFT (frame length 25 ms, hop size 10 ms), with 80 Mel bins covering 0–8 kHz. Let the linear-scale Mel energy be $p(t, f)$. To remove leading/trailing silence, frame energy is computed as

$$E_t = \sum_{f=1}^{F} P(t, f) \tag{S1}$$

where $f = 80$. Frames below a preset energy threshold are trimmed from both ends.

S1.2 Spectral gating noise reduction

Let $X(t, f)$ denote the log-Mel spectrogram:

$$X(t, f) \in \mathbb{R}^{T \times F} \tag{S2}$$

We map log-Mel to linear energy:

$$P(t, f) = \exp\big(X(t, f)\big) \tag{S3}$$

To estimate the time-varying noise floor per frequency bin, a sliding low-quantile statistic is used. Let $\mathcal{W}_t = \{t - w, \ldots, t + w\}$ be a temporal window centered at t with half-width w. The noise estimate is defined as

$$\tilde{N}(t, f) = Q_q\big(\{P(t', f) \mid t' \in \mathcal{W}_t\}\big) \tag{S4}$$

where $Q_q(\cdot)$ is the quantile operator. In this work, we set $q = 0.20$ and $w = 25$ frames (window length $2w + 1 = 51$ frames, approximately 0.51 s with a 10 ms hop).

An adaptive threshold is then constructed as

$$\Theta(t, f) = \gamma \tilde{N}(t, f) + \beta \tag{S5}$$

Where γ and β control suppression strength and avoid over-suppression under extremely low-noise conditions. In this work, $\gamma = 1.20$ and $\beta = 0.20$.

A soft gain mask is computed as

$$G_0(t, f) := \max\left(g_{\min}, \frac{P(t, f) - \lambda\Theta(t, f)}{P(t, f) + \delta} \right) \tag{S6}$$

where λ is an over-subtraction factor, δ is a numerical-stability constant, and $g_{\min}$ is a gain floor.

To mitigate musical-noise artifacts, the gain mask is smoothed and clipped:

$$G(t, f) = \max\left(g^S, \mathcal{S}\left(G_0(t, f)\right)\right) \tag{S7}$$

where $S(\cdot)$ is a 2D smoothing operator and η is a post-smoothing floor.

Finally, the gated log-Mel feature is obtained by

$$\tilde{X}(t, f) = \log\left(G(t, f)P(t, f) + \epsilon\right) \tag{S8}$$

where ϵ is a stability constant. The gated feature $\tilde{X}$ is used as the input to ATF-Conformer.

To explicitly define the operation as a preprocessing mapping (used for reproducible implementation), we summarize the full spectral-gating transform as

$$\tilde{X} = \mathcal{G}\left(X; q, w, \gamma, \beta, \lambda, \delta, g_{\min}, g^S, \eta, \epsilon\right) \tag{S9}$$

where $\mathcal{G}(\cdot)$ denotes the deterministic spectral-gating operator specified by Eqs. (S3)–(S8).

Implementation note (for reproducibility). Parameters $\lambda, \delta, g_{\min}, S(\cdot)$ (and kernel size), η, and ϵ are implementation constants used for stability and smoothing and were fixed across all experiments (i.e., not tuned on validation/test sets). The exact values follow the released implementation.

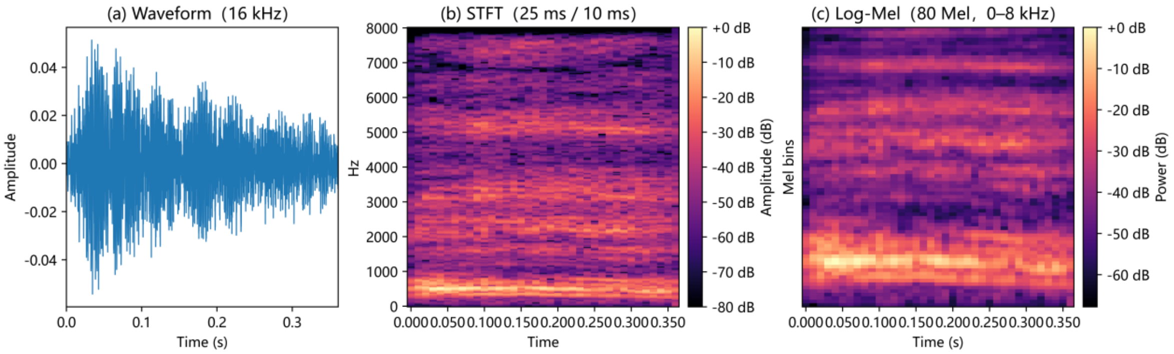

Figure S1. Audio feature extraction and preprocessing pipeline.

Overview of the preprocessing pipeline: log-Mel extraction (16 kHz; 25 ms/10 ms; 80 Mel bins, 0–8 kHz) → silence trimming → spectral gating (Eqs. (S3)–(S9)) → gated log-Mel $\tilde{X}$ for ATF-Conformer.

Algorithm S1. Forward propagation of ATF-Conformer

Algorithm S1. Forward propagation of ATF-Conformer

Input: log-Mel spectrogram $\tilde{X}$ (or gated $\tilde{X}$ from Section S1)

Output: predicted label $\hat{y}$

1. $H_c \leftarrow \mathrm{Conv}(X)$
2. For $d \in CT, CF, TF\$:\$H_d \leftarrow a_d(H_c)^\circ H_c$
3. $H_{TA} \leftarrow \frac{1}{3}(H_{CT} + H_{CF} + H_{TF})$
4. $H_T \leftarrow \mathrm{MHSA}(H_{TA})$
5. $H_F \leftarrow \mathrm{Conv}_{1\times k}(H_T A)$
6. $H \leftarrow \mathrm{FFN}([H_T; H_F])$
7. $\alpha \leftarrow \mathrm{softmax}(WH)$
8. $z \leftarrow \sum_{t=1}^{T} m_t \alpha_t h_t$
9. Compute class logits from z, and output. $\hat{y} \leftarrow \mathrm{argmax}, \mathrm{softmax}(\mathrm{logits})$
10. return $\hat{y}$
11. End Procedure
